# A reactive oxygen species activation mechanism contributes to JS-K-induced apoptosis in human bladder cancer cells

**DOI:** 10.1038/srep15104

**Published:** 2015-10-13

**Authors:** Mingning Qiu, Lieqian Chen, Guobin Tan, Longzhi Ke, Sai Zhang, Hege Chen, Jianjun Liu

**Affiliations:** 1 Laboratory of Urology, Guangdong Medical University, Zhanjiang 524001, China

## Abstract

Reactive oxygen species (ROS) and cellular oxidant stress are regulators of cancer cells. The alteration of redox status, which is induced by increased generation of ROS, results in increased vulnerability to oxidative stress. The aim of this study is to investigate the influence of O2-(2,4-dinitrophenyl) 1-[(4-ethoxycarbonyl)piperazin-1-yl]diazen-1-ium-1,2-diolate (JS-K, C_13_H_16_N_6_O_8_) on proliferation and apoptosis in bladder cancer cells and explored possible ROS-related mechanisms. Our results indicated that JS-K could suppress bladder cancer cell proliferation in a concentration- and time-dependent manner and induce apoptosis and ROS accumulation in a concentration-dependent manner. With increasing concentrations of JS-K, expression of proteins that are involved in cell apoptosis increased in a concentration-dependent manner. Additionally, the antioxidant N-acetylcysteine (NAC) reversed JS-K-induced cell apoptosis; conversely, the prooxidant oxidized glutathione (GSSG) exacerbated JS-K-induced cell apoptosis. Furthermore, we found that nitrites, which were generated from the oxidation of JS-K-released NO, induced apoptosis in bladder cancer cells to a lower extent through the ROS-related pathway. In addition, JS-K was shown to enhance the chemo-sensitivity of doxorubicin in bladder cancer cells. Taken together, the data suggest that JS-K-released NO induces bladder cancer cell apoptosis by increasing ROS levels, and nitrites resulting from oxidation of NO have a continuous apoptosis-inducing effect.

Nitric oxide (NO) is a major signaling molecule, toxicant, and antioxidant under many conditions. NO is involved in various physiological and pathological processes. NO donor drugs have been reported to induce apoptosis in several types of human cancer cells.^1^,^2^. O2-(2,4-Dinitrophenyl) 1-[(4-ethoxycarbonyl)piperazin-1-yl]diazen-1-ium-1,2-diolate (JS-K, C_13_H_16_N_6_O_8_, CAS-No.: 205432-12-8) is a diazeniumdiolate-based NO-donor prodrug and is reportedly highly cytotoxic to human cancer cells such as acute myeloid leukemia^3^, multiple myeloma^4^, non-small-cell lung cancer^5^, malignant glioma^6^, breast cancer^7^ and prostate cancer cells^8^ and to murine erythroleukemia cells^9^. As a glutathione/glutathione S-transferase-activated nitric oxide donor, JS-K selectively exhibits potent antitumor activity against human cancer cells^3^,^8^ and has no significant toxicity toward normal cells.

Reactive oxygen species (ROS) are signaling molecules generated by mitochondria that participate in stress signaling in normal cells. ROS also activate intracellular signal transduction pathways that regulate multiple events in cancer, such as inflammation, cell cycle progression, apoptosis, migration and invasion^10^,^11^. Previous studies have reported increased generation of ROS in cancer cells and that alteration of the redox status causes cells to be more vulnerable to increased oxidative stress induced by exogenous ROS-generating compounds^12^. As a NO-donor prodrug, JS-K is reported to inhibit cancer cell proliferation and induce apoptosis^9^, and treatment with JS-K results in oxidative/nitrosative stress in non-small-cell lung cancer cells^5^. In this study, JS-K promoted ROS levels, increased cytotoxicity and caspase-3/7 activity, and activated caspase-9 protein in bladder cancer cells in a concentration-dependent manner; these effects, in turn, induced cellular apoptosis. Treatment with the antioxidant N-acetylcysteine (NAC) reversed JS-K-induced cell growth suppression and apoptosis, while treatment with the prooxidant oxidized glutathione (GSSG) exacerbated the effects of JS-K. In addition, JS-K-released NO was oxidized into nitrites, which subsequently induced apoptosis in bladder cancer cells through an ROS-related pathway.

## Results

### JS-K suppressed proliferation and induced apoptosis in bladder cancer cells

**Figure 1:**
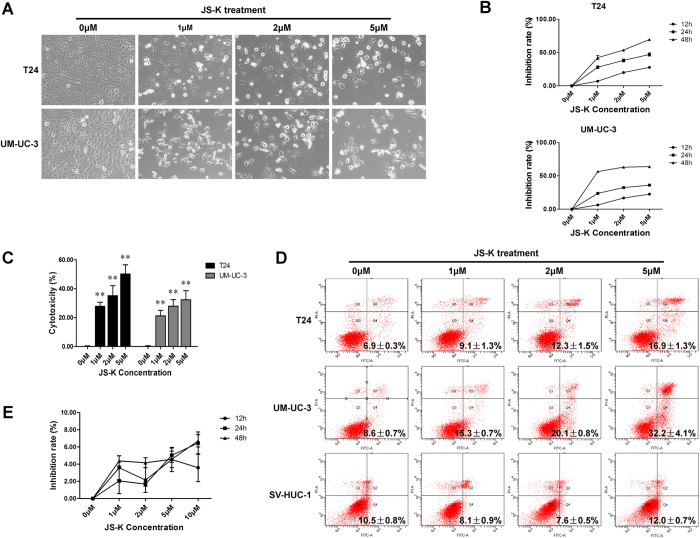
JS-K inhibits cell proliferation and promotes cell apoptosis. (**A**) JS-K-induced apoptosis in T24 and UM-UC-3 cells at 24 h as visualized by microscopy (100×). (**B**) Cell proliferation, as detected by the CCK-8 assay, was suppressed in a concentration- and time-dependent manner after JS-K treatment. (**C**) Cytotoxicity of JS-K was determined by an LDH assay. The data indicate that JS-K affected bladder cancer cells in a concentration-dependent manner. (**D**) JS-K-induced cell apoptosis was analyzed by flow cytometry. After 24 h treatment with JS-K, apoptosis of T24, UM-UC-3 and SV-HUC-1 cells were measured, and the data indicate that JS-K induced apoptosis of T24 and UM-UC-3 but had no significant effect on SV-HUC-1 cells. (**E**) JS-K did not inhibit cell proliferation in SV-HUC-1 human nephric tubule cells. The data are presented as the mean ± SD for at least three independent experiments. Double asterisks (**) indicate an extremely significant difference (*P* < 0.01).

Bladder cancer cells were exposed to various concentrations (1 μM, 2 μM and 5 μM) of JS-K. We found that untreated cells grew well, whereas cells treated with JS-K for 24 h were distorted in shape, became round and underwent apoptosis (Fig. 1A). A CCK-8 assay was performed to evaluate the effects of JS-K on bladder cancer cells, and the data indicated that JS-K inhibited growth of T24 and UM-UC-3 cells in a concentration- and time-dependent manner (Fig. 1B). The IC50 concentrations were 1.59 ± 0.11 μM (T24 cells) and 0.52 ± 0.04 μM (UM-UC-3 cells) at 48 h. These data revealed that JS-K could significantly reduce the viability of bladder cancer cells. The apoptosis-inducing effect and cytotoxicity of JS-K were analyzed using a FITC Annexin V Apoptosis Detection Kit and an LDH Cytotoxicity Assay Kit, respectively. Treatment with JS-K for 24 h increased apoptosis (Fig. 1D) and cytotoxicity (Fig. 1C) in bladder cancer cells in a concentration-dependent manner. These results indicated that JS-K significantly suppressed proliferation and induced apoptosis of T24 and UM-UC-3 cells in a concentration-dependent manner. In contrast, cells of the human nephric tubule cell line SV-HUC-1 were not sensitive to JS-K (Fig. 1D,E).

### JS-K increased ROS production and reduced the GSH/GSSG ratio in bladder cancer cells

**Figure 2:**
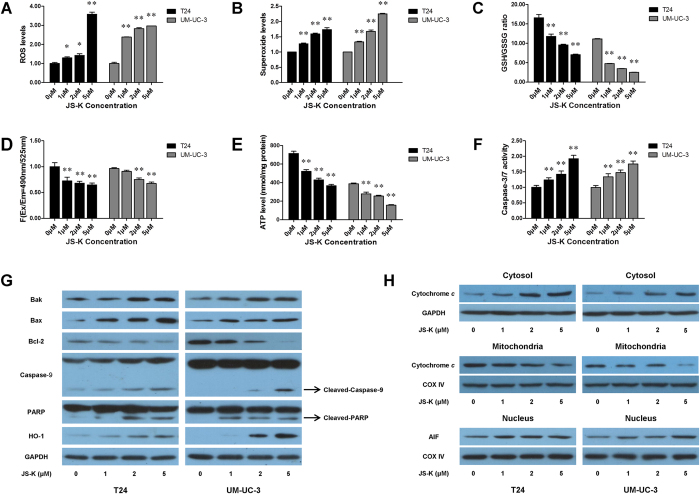
Concentration-dependent effect of JS-K on ROS, GSH/GSSG ratio, mitochondrial membrane potential, ATP production and apoptotic proteins. Bladder cancer cells were treated with JS-K (0 μM, 1 μM, 2 μM and 5 μM) for 6 h, and then the intracellular level of total ROS (**A**), the level of superoxide (**B**), the GSH/GSSG ratio (**C**), the mitochondrial membrane potential (**D**), ATP production (**E**) and caspase-3/7 activity (**F**) were detected. The ROS levels and caspase-3/7 activity in JS-K-treated cells were significantly increased compared with their levels in the vehicle control. The GSH/GSSG ratio, mitochondrial membrane potential, and ATP production were markedly decreased. (**G**) Cells were treated with JS-K (0 μM, 1 μM, 2 μM and 5 μM) for 24 h, and the levels of Bak, Bax, Bcl-2, caspase-9, PARP and HO-1 proteins were examined by western blot. The data indicate that JS-K regulated apoptotic proteins in bladder cancer cells in a concentration-dependent manner. (**H**) Cytochrome *c* was detected in the mitochondria and cytosol, and AIF was detected in the nucleus. The release of cytochrome *c* from the mitochondria to the cytosol was demonstrated. The AIF level was upregulated in JS-K-treated cells. The data are presented as the mean ± SD for at least three independent experiments. Single asterisks (*) indicate a significant difference (*P* < 0.05), and double asterisks (**) indicate an extremely significant difference (*P* < 0.01).

We measured the total ROS production and superoxide level in T24 and UM-UC-3 cells that were treated with various concentrations of JS-K for 6 h. Treatment with JS-K resulted in significantly increased production of ROS in T24 and UM-UC-3 cells (Fig. 2A,B). We also detected levels of GSH and GSSG and found that the GSH/GSSG ratio was significantly decreased (Fig. 2C). The imbalance of ROS and the GSH/GSSG ratio may promote mitochondrial dysfunction and lead to mitochondria-mediated apoptosis.

### JS-K decreased mitochondrial membrane potential and ATP levels in bladder cancer cells

**Figure 3:**
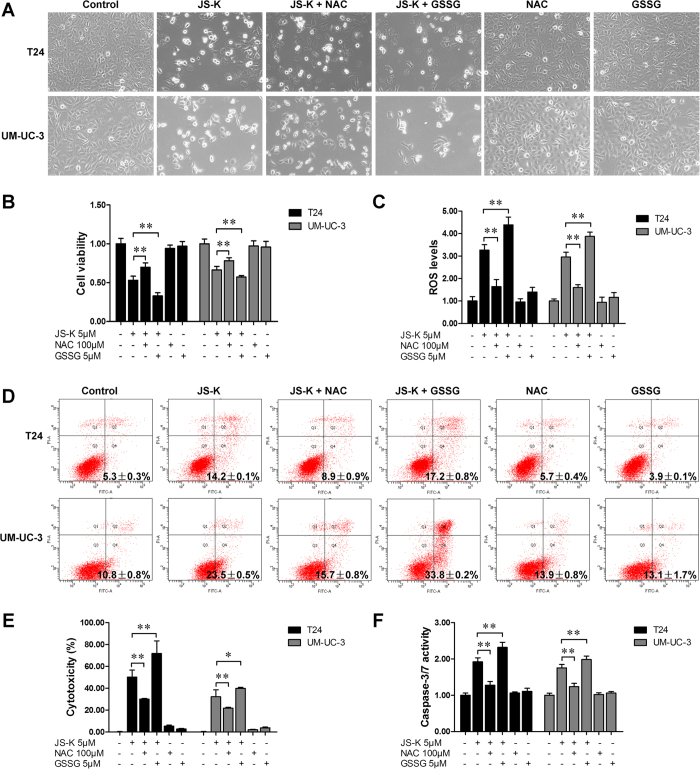
Effects of NAC and GSSG on JS-K-induced cell apoptosis. (**A**) JS-K-induced apoptosis in T24 and UM-UC-3 cells with or without NAC/GSSG. T24 and UM-UC-3 cells were cultured with 100 μM NAC or 5 μM GSSG for 24 h, and then treated with or without 5 μM JS-K. The cells were visualized by microscopy (100×). (**B**) Cell survival was detected by CCK-8 assay. (**C**) Cells were pretreated with 100 μM NAC or 5 μM GSSG for 24 h and subsequently treated with or without 5 μM JS-K for 6 h, ROS production was then measured. (**D**) Apoptosis was analyzed according to the distributions for the treatments in (**A**). Cytotoxicity (**E**) and caspase-3/7 activity (**F**) were determined according to the distributions for the treatments in (**A**). The data are presented as the mean ± SD for at least three independent experiments. Single asterisks (*) indicate a significant difference (*P* < 0.05), and double asterisks (**) indicate an extremely significant difference (*P* < 0.01).

To evaluate the dysfunction in mitochondrial energy production, we measured the mitochondrial membrane potential and intracellular levels of ATP in JS-K-treated bladder cancer cells. T24 and UM-UC-3 cells were treated with various concentrations of JS-K for 6 h, and the results indicated that the mitochondrial membrane potential (Fig. 3D) and intracellular levels of ATP (Fig. 3E) decreased in a concentration-dependent manner.

### JS-K regulated the production of apoptotic proteins in bladder cancer cells

Increased levels of ROS induce cell apoptosis by releasing the pro-apoptotic protein cytochrome *c*, which is released from the mitochondria into the cytosol. In this study, T24 and UM-UC-3 cells were treated with various concentrations of JS-K for 24 h, and cytochrome *c* was found to be increased in the cytosol but decreased in the mitochondria (Fig. 3H). Apoptosis-inducing factor (AIF) was also measured and found to be upregulated in the nucleus (Fig. 3H). Increased activity of caspase-9 and caspase-3/7 and increased levels of cleaved PARP were detected in JS-K-treated bladder cancer cells compared with the controls (Fig. 3F,G). The oxidative stress-related protein HO-1 and pro-apoptotic proteins Bak and Bax were upregulated, whereas Bcl-2 was downregulated in a concentration-dependent manner (Fig. 3G).

### NAC reversed JS-K-induced cell growth suppression and apoptosis, and GSSG exacerbated the effects of JS-K

To determine the roles of ROS in JS-K-induced cell growth suppression and apoptosis, T24 and UM-UC-3 cells were treated with JS-K in the presence or absence of the antioxidant NAC (100 μM) or pro-oxidant GSSG (5 μM). As shown in Fig. 3, NAC reversed JS-K-induced T24 and UM-UC-3 cell growth suppression and apoptosis and recovered ROS levels to a great extent; GSSG exacerbated JS-K-induced cell growth inhibition and apoptosis and promoted JS-K-mediated ROS production.

### JS-K-released NO was oxidized into nitrites, which continued to induce apoptosis in bladder cancer cells

**Figure 4:**
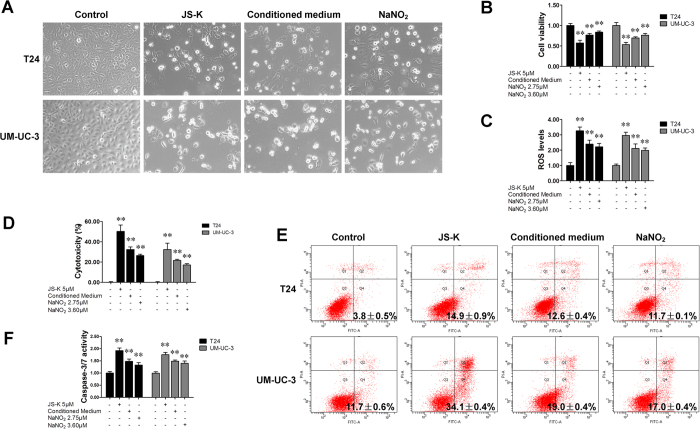
JS-K released nitrites and continued to induce apoptosis in bladder cancer cells. (**A**) Apoptosis in T24 and UM-UC-3 cells treated with JS-K, conditioned medium or NaNO_2_. T24 and UM-UC-3 cells were cultured with either the conditioned medium of cells treated with JS-K (5 μM) or with NaNO_2_ (2.75 μM for T24 and 3.60 μM for UM-UC-3) for 48 h. Cells treated with 5 μM JS-K were used as a positive control. The cells were visualized by microscopy (100×). (**B**) Cell survival was assessed using a CCK-8 assay. (**C**) Cells were treated with JS-K (5 μM), conditioned medium or NaNO_2_ (2.75 μM for T24 and 3.60 μM for UM-UC-3) for 6 h. ROS production was then measured. Cytotoxicity (**D**), apoptosis (**E**) and caspase-3/7 activity (**F**) were determined according to the distributions for the treatments in (**A**). The data are presented as the mean ± SD for at least three independent experiments. Double asterisks (**) indicate an extremely significant difference (*P* < 0.01).

**Figure 5:**
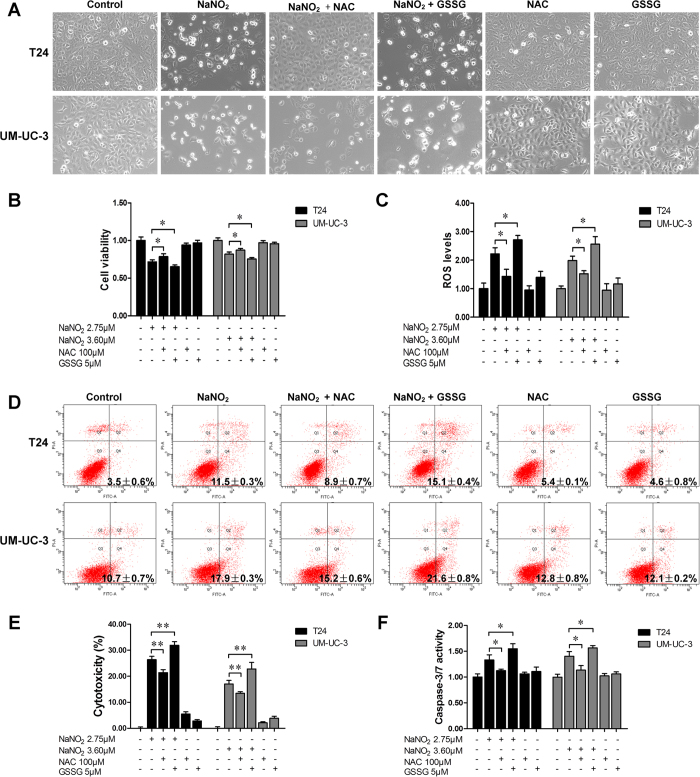
Effects of NAC and GSSG on NaNO_2_-induced cell growth suppression and apoptosis. (**A**) NaNO_2_-induced apoptosis in T24 and UM-UC-3 cells. T24 and UM-UC-3 cells were pretreated with 100 μM NAC or 5 μM GSSG for 24 h and then treated with or without NaNO_2_ (2.75 μM for T24 and 3.60 μM for UM-UC-3). The cells were visualized by microscopy (100×). (**B**) Cell survival assay. (**C**) After pretreatment with 100 μM NAC or 5 μM GSSG for 24 h, cells were treated with or without NaNO_2_ for 6 h. The ROS level was then determined. Apoptosis (**D**), cytotoxicity (**E**) and caspase-3/7 activity (**F**) were analyzed according to the distributions for the treatments in (**A**). The data are presented as the mean ± SD for at least three independent experiments. Single asterisks (*) indicate a significant difference (*P* < 0.05), and double asterisks (**) indicate an extremely significant difference (*P* < 0.01).

Nitric oxide will oxidize into a nitrite ion when exposed to oxygen. To further study the cytotoxic roles of JS-K, the levels of nitrite ion (NO_2_^−^) in the culture media of T24 and UM-UC-3 cells incubated with JS-K (5 μM) were determined and were found to be 2.74 ± 0.19 μM (in T24 cells and 3.62 ± 0.13 μM in UM-UC-3 cells. The effects of nitrite ions were then analyzed using the conditioned medium of JS-K-treated cells (5 μM, 24 h cultured) and using NaNO_2_ (2.75 μM/3.60 μM). The data indicated that the conditioned medium of JS-K-treated cells and NaNO_2_ were also able to induce apoptosis in T24 and UM-UC-3 cells, although to a smaller extent with JS-K-conditioned medium (Fig. 4). Results similar to those obtained with JS-K treatment were achieved when cells were pre-treated with NAC or GSSG and then subsequently treated with conditioned medium (Figure S1) or with NaNO_2_ (Fig. 5).

### JS-K enhanced the chemo-sensitivity of doxorubicin in bladder cancer cells

**Figure 6:**
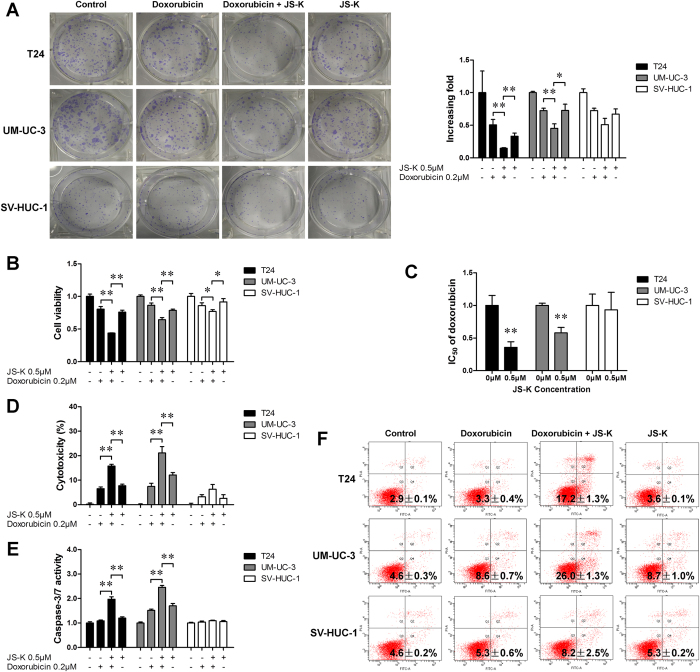
JS-K enhanced the chemo-sensitivity of doxorubicin in bladder cancer cells. (**A**) Colony formation assay with different treatments of JS-K and doxorubicin in bladder cancer cells. Representative graphs (left). The clones were quantified, and the results are presented in a graph (right). (**B**) Cell viability was measured by a CCK-8 assay after treatment with JS-K and doxorubicin. (**C**) Changes in the IC50 of doxorubicin. (**D**) Cytotoxicity of different drug combinations. (**E**) Caspase-3/7 activity of bladder cancer cells with various drug concentrations and treatment durations. (**F**) Apoptosis of T24 and UM-UC-3 cells induced by the drugs individually and in combination at different concentrations and treatment durations. The data are presented as the mean ± SD for at least three independent experiments. Single asterisks (*) indicate a significant difference (*P* < 0.05), and double asterisks (**) indicate an extremely significant difference (*P* < 0.01).

To evaluate whether JS-K could improve the chemo-sensitivity of doxorubicin, JS-K and doxorubicin were administered together to bladder cancer cells. As shown in Fig. 6, the combination of JS-K and doxorubicin showed a greater effect on the inhibition of colony formation ability (Fig. 6A) and cell viability (Fig. 6B) than the use of JS-K or doxorubicin individually. Notably, JS-K decreased the IC_50_ of doxorubicin in T24 and UM-UC-3 cells (Fig. 6C). The combination of JS-K and doxorubicin showed a synergistic effect on the promotion of tumor cell apoptosis (Fig. 6F). JS-K also increased the cytotoxicity of doxorubicin (Fig. 6D) and doxorubicin-induced caspase-3/7 activity (Fig. 6E).

## Discussion

It is widely recognized that reactive oxygen species (ROS) play critical roles in causing cell damage and physiological dysfunction and that the accumulation of ROS is involved in multiple events in cancer, including inflammation, cell cycle progression, apoptosis, migration, and invasion^13^,^14^. In the present study, we demonstrated that JS-K could induce apoptosis in bladder cancer cells. As the concentration of JS-K increased, cell growth decreased and apoptosis increased. However, JS-K had no detectable effect on cell proliferation or apoptosis in human nephric tubule SV-HUC-1 cells. A previous study demonstrated that the nitric oxide prodrug JS-K is effective against non-small cell lung cancer cells and acts by increasing intracellular ROS levels^5^, and our data are consistent with this result. Furthermore, we showed that JS-K-released NO is oxidized into nitrites, which induced apoptosis in bladder cancer cells. In addition, JS-K was shown to improve the chemo-sensitivity of doxorubicin in bladder cancer cells.

Various physiological phenomena and chemicals that are considered to be able to induce cell apoptosis are known to provoke oxidative stress by generating ROS, which suggests a close relationship between oxidative stress and apoptosis^15^,^16^. Under pathological conditions, the redox state can be altered to lower or higher values, whereas it is maintained within a narrow range under normal conditions^17^. In the present study, JS-K was found to dramatically increase the production of ROS in bladder cancer cells in a concentration-dependent manner, which might disrupt the balance of redox reactions. We also found that treatment with JS-K induced apoptosis in bladder cancer cells, and GSSG exacerbated the JS-K-induced apoptosis. However, the pro-apoptotic effect was reversed with the antioxidant NAC. These results suggest that the balance between ROS production, antioxidants and prooxidants is very important for the proliferation of bladder cancer cells.

In recent years, a physiologically balanced redox state, such as GSH/GSSG, has been used to describe a general cellular oxidation/reduction environment^18^. GSH is recognized as an important antioxidant that is associated with mitochondrial dysfunction and cell apoptosis, and GSH depletion might lead to redox imbalance^19^. In our study, JS-K significantly reduced the production of GSH and increased the production of GSSG in bladder cancer cells, which resulted in a decline of the GSH/GSSG ratio. Moreover, ATP functions as a direct energy source for cellular metabolism, and the amount of ATP may be affected by a decrease in the mitochondrial membrane potential during apoptosis^5^. Our results showed that JS-K significantly decreased the GSH/GSSG ratio, the generation of ATP, and the mitochondrial membrane potential in a concentration-dependent manner. Heme oxygenase-1 (HO-1) is a widespread and rate-limiting enzyme that catabolizes heme to carbon monoxide (CO), ferrous iron, and biliverdin^20^. HO-1 is induced by many stresses such as oxidative stress and hypoxia-ischemia and plays important roles in maintaining cellular homeostasis^20^,^21^. Previous studies have demonstrated that elevated HO-1 levels affect tumorigenesis and tumor growth^22^,^23^. In various tissues, the induction of HO-1 mRNA serves as an essential biomarker of cellular oxidative stress^24^ a and is assumed to play important roles in the induction of chemotherapeutic protective mechanisms^25^. HO-1 is always expressed at a low level under normal conditions but is highly induced in response to various reagents that cause oxidative stress. In a study in 2011, HO-1 was reported to be downregulated after JS-K treatment in breast cancer cells^7^. However, in our study, we found that JS-K treatment upregulated HO-1 expression and resulted in apoptosis in bladder cancer cells in a concentration-dependent manner. These conflicting results might be due to the difference of tissues.

We performed western blot analysis of cytochrome *c* and AIF in bladder cancer cells. The results indicated that a mitochondria-dependent cell apoptosis pathway is involved in post-mitochondrial pharmacological and possibly endogenous regulation of the caspase-independent death effector AIF. We observed increased AIF levels in the nucleus, and we also found that cytochrome *c* released from the mitochondria to the cytosol had a pro-apoptotic effect by activating caspase-9. As a substrate of caspase-9, PARP appears to be involved in DNA repair in response to environmental stress^26^. The activation of caspase-9 is a crucial step during the process of apoptosis because caspase-9 induces PARP cleavage^27^. PARP is proposed to help cells maintain their viability; however, cleavage of PARP facilitates cellular disassembly and is recognized as an early DNA damage response indicator and a marker of cells undergoing apoptosis^28^. Previous studies have demonstrated that maintaining the balance between Bcl-2 and Bax is crucial for cell survival and that a higher Bcl-2 level results in suppression of apoptosis^29^. In this study, we confirmed that JS-K increases levels of cleaved caspase-9 and PARP proteins in bladder cancer cells but decreases the level of Bcl-2 protein. Caspase-3 and caspase-7 are also critical contributors to the execution of apoptosis, as they are either partially or totally responsible for the cleavage of PARP^30^. The results obtained in the Caspase-Glo 3/7 assay demonstrated that JS-K increased caspase-3/7 activity in bladder cancer cells. These data suggest that increased ROS-mediated signaling may promote apoptosis in human bladder cancer cells.

NO is reported to induce DNA damage in human cancer cells by stimulating ROS and RNS, which play a key role in human cancers^31^,^32^. NO and nitrites are also suggested to inhibit activity of OGG1, a protein involved in the repair of 8-oxoguanine, thereby inducing DNA damage^33^. In a recent study in prostate cancer cells, the maximal level of NO generation by JS-K was reported to be detectable after nine to twelve hours^8^. We detected a maximal level of NO generation by JS-K over a similar time window (Figure S2). However, in the present study, we found that bladder cancer cells continued to undergo apoptosis until 48 hours after JS-K treatment despite there being no more NO released in the culture medium. The bladder cancer cells had been allowed to recover for up to 36 h in fresh medium after withdrawal of JS-K, with which the cells had been treated for 12 hours (Figure S3). Thus, we speculated that JS-K released NO that was oxidated into nitrites, which subsequently played roles in bladder cancer cells. We investigated the effect of nitrites on ROS levels and apoptosis in bladder cancer cells and found similar results as we had found with JS-K treatment. These results suggest that nitrites from NO sources continued to induce apoptosis in bladder cancer cells.

**Figure 7:**
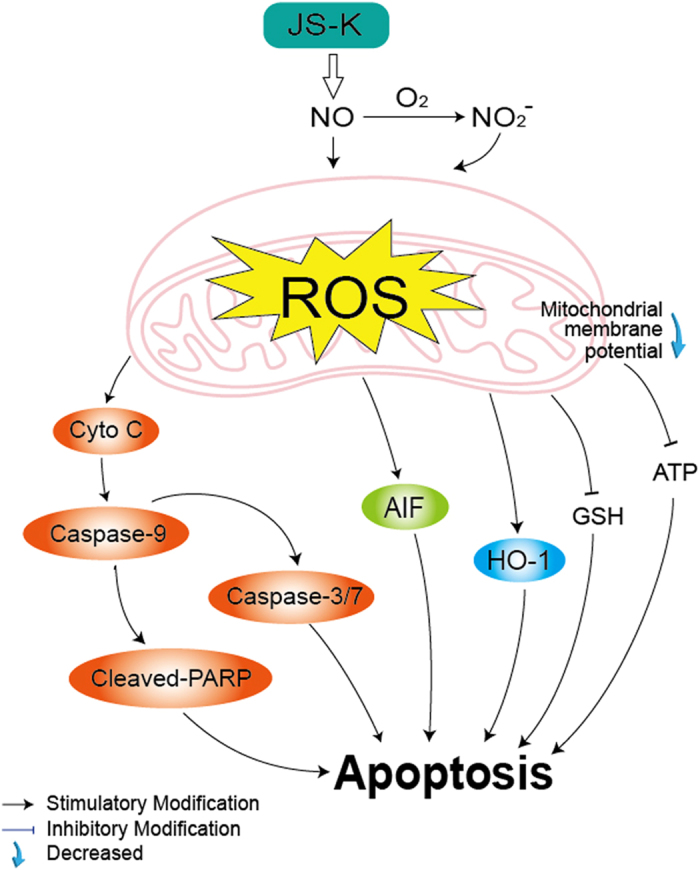
Cellular pathway of JS-K-induced cell apoptosis in bladder cancer cells. JS-K-induced bladder cancer cell apoptosis via the activation of cytochrome *c*, caspase-3/7 and AIF suppression of mitochondrial membrane potential and ATP. The production of ROS increased, which eventually led to apoptosis.

In summary, redox imbalance due to excessive or insufficient ROS is a critical induction factor for cancer development and progression. We confirmed that ROS are significantly associated with bladder cancer cell proliferation and that excessive ROS could stimulate a mitochondria-dependent cell apoptosis pathway, leading to apoptosis. In the present study, we found that apoptosis that was induced by JS-K could be reversed by an antioxidant. We hypothesize that an imbalance in redox reactions occurred to induce apoptosis in bladder cancer cells (Fig. 7). The influence of nitrites from NO sources on bladder cancer cells was also investigated, and effects similar to those obtained with JS-K treatment were observed. Although further investigations should be performed to determine the molecular mechanism underlying the JS-K-induced generation of ROS, our data suggest that JS-K-induced apoptosis may act, at least partially, through an ROS-related pathway in bladder cancer cells.

## Methods

### Drugs and reagents

The nitric oxide prodrug JS-K was purchased from Santa Cruz Biotechnology, dissolved in 100% DMSO to a concentration of 5 M as a stock solution, and stored at −20 °C. The final concentration of DMSO did not exceed 0.1% throughout the study. N-acetylcysteine (NAC) and oxidized glutathione (GSSG) were purchased from Beyotime Institute of Biotechnology (Shanghai, China) and dissolved in PBS to concentrations of 100 mM (NAC) and 10 mM (GSSG). Antibodies to poly ADP-ribose polymerase (PARP), caspase-9, Bak, Bcl-2, Bax, cytochrome *c*, heme oxygenase 1 (HO-1) and apoptosis-inducing factor (AIF) were obtained from Cell Signaling Technology, and an antibody to glyceraldehyde-3-phosphate dehydrogenase (GAPDH) was purchased from Abcam to be used as a loading control. The goat anti-rabbit IgG-HRP secondary antibody was purchased from EarthOx (USA).

### Cell culture and JS-K treatment

Human bladder cancer cell lines T24 and UM-UC-3 and human nephric tubule cell line SV-HUC-1 were purchased from Guangzhou Jennio Biological Technology Co., Ltd. (Guangzhou, China) and were cultured in high glucose DMEM medium (GIBCO) supplemented with 10% (v/v) fetal bovine serum (FBS, GIBCO), 100 U/mL penicillin, and 100 U/mL streptomycin at 37 °C under an atmosphere of 5% CO_2_ in humidified air.

After 24 h in culture, bladder cancer cells were treated with different concentrations (1 μM, 2 μM and 5 μM) of JS-K for 12 h, 24 h and 48 h. NAC and GSSG were dissolved in medium (NAC: 100 μM; GSSG: 5 μM) and were incubated with JS-K (5 μM) for 24 h.

### Conditioned media collection and subsequent treatment

The liquid supernatants of culture media were collected as conditioned media by centrifuging T24 and UM-UC-3 cells at 2500 r/min for 15 min after the cells had been treated with JS-K (5 μM) for 24 h. The concentrations of nitrate (NO_2_^−^) in conditioned media were examined with a Nitrite Assay Kit (Beyotime). T24 and UM-UC-3 cells were subsequently treated with the conditioned media or with similar concentrations of NaNO_2_ in fresh culture medium for 48 h. The cells treated with JS-K (5 μM) for 24 h were defined as a positive control.

### CCK-8 assay

Cell proliferation was detected with a Cell Counting Kit-8 (CCK-8, Dojindo, Japan) according to the manufacturer’s instructions. Briefly, cells were seeded into 96-well plates (Corning) at a density of 10^4^ cells per well in 100 μL culture medium. After treating with JS-K, conditioned medium, or NaNO_2_, the culture medium was removed and replaced with 100 μL medium containing CCK-8 reagent (CCK-8: 10 μL, Dojindo, Japan) and then incubated at 37 °C for 2 h. The absorbance was recorded at a wavelength of 450 nm in a 96-well plate reader (EnSpire 2300 Multilabel Reader, PE).

### Apoptosis detection

Apoptotic cells were quantified using the FITC Annexin V Apoptosis Detection Kit (BD Pharmingen, USA) according to the manufacturer’s protocols. Briefly, after treatment with JS-K, conditioned medium, or NaNO_2_, the cells were collected, resuspended in binding buffer, and incubated with Annexin V-FITC and PI for 15 min at room temperature in the dark. The stained cells were analyzed by flow cytometry within 1 h.

### Caspase-Glo 3/7 assays

To examine cell apoptosis after treatment, caspase-3/7 activity was analyzed by Caspase-Glo 3/7 assays in accordance with the manufacturer’s protocol. Briefly, cells were seeded into 96-well dishes (Corning) and exposed to JS-K, conditioned medium, or NaNO_2_. Then, an equal volume of Caspase-Glo 3/7 reagent was added into each well, and the cells were incubated for 30 min at room temperature in the dark. The luminescence was measured by a luminometer (Berthold Sirius L, Germany).

### Cytotoxicity assay

The cytotoxicity of JS-K, conditioned medium, or NaNO_2_ was analyzed with an LDH Cytotoxicity Assay Kit (Beyotime) according to the manufacturer’s protocol. Briefly, cells were cultured in 96-well plates (Corning) and treated with JS-K, conditioned medium, or NaNO_2_. The culture media were centrifuged at 400 × *g* for 5 min. The supernatants (120 μL/well) were transferred into a corresponding 96-well plate, and 60 μL of LDH detection reagent was added to each well. The plates were then incubated for 30 min at room temperature in the dark. The absorbance was recorded at 490 nm with a reader (EnSpire 2300 Multilabel Reader, PE). The percentage cytotoxicity was calculated as follows: Cytotoxicity (%) = (Absorbance of Test Sample – Absorbance of Low Control)/(Absorbance of Maximum Enzyme Activity – Absorbance of Low Control) × 100.

### Measurement of intracellular ROS level

Accumulation of intracellular ROS in bladder cancer cells was quantified with a Reactive Oxygen Species Assay Kit (Beyotime). After treatment with JS-K (1 μM, 2 μM and 5 μM), conditioned medium, or NaNO_2_ for 6 h, cells were collected and resuspended with serum-free medium containing DCFH-DA (10 μM). Subsequently, cells were incubated at 37 °C for 20 min in the dark. DCF fluorescence intensity was measured by flow cytometry with the excitation source at 488 nm and emission at 525 nm.

### Intracellular glutathione content assay

T24 and UM-UC-3 cells were plated in 6-well plates and treated with various concentrations of JS-K (1 μM, 2 μM and 5 μM) for 6 h. After treatment, cells were harvested and lysed by two successive rounds of freezing and thawing. The precipitate was removed by centrifuging at 10,000 × *g* for 10 min, and the supernatant was analyzed for GSH and glutathione disulfide (GSSG) levels using a GSH and GSSG Assay Kit (Beyotime) according to the manufacturer’s protocol.

### Intracellular superoxide measurement

Intracellular superoxide levels were measured with a Superoxide Assay Kit (Beyotime) according to the manufacturer’s protocol. Briefly, cells were seeded into 96-well dishes (Corning) and treated with JS-K (1 μM, 2 μM and 5 μM) for 6 h. The culture medium containing JS-K was replaced with fresh medium, and the cells were washed with PBS. Superoxide detection reagent was added into each well (200 μL/well) and incubated at 37 °C for 20 min. The absorbance was recorded at 450 nm in a 96-well plate reader (EnSpire 2300 Multilabel Reader, PE).

### Measurement of mitochondrial membrane potential

T24 and UM-UC-3 cells were seeded into 6-well plates at 3 × 10^5^/well and allowed to grow for 24 h, then were treated with different concentrations of JS-K (1 μM, 2 μM and 5 μM) for 3 h. The JC-1 Mitochondrial Membrane Potential Assay Kit (Beyotime) was used according to the manufacturer’s instructions. The cells were analyzed by flow cytometry.

### Measurement of ATP production

ATP levels were measured using an ATP Assay Kit (Beyotime) according to the manufacturer’s instructions. Briefly, bladder cancer cells were treated with JS-K (1 μM, 2 μM and 5 μM) for 6 h and incubated in 200 μL lysis buffer at 4 °C. The supernatant was then collected by centrifuging at 12,000 × *g* for 10 min at 4 °C. Subsequently, 50 μL of supernatant was added to 100 μL ATP detection reagent, and firefly luciferase activity was detected using a luminometer (Berthold Sirius L, Germany).

### Colony formation assay

Cells were pre-treated with drugs for 24 h. Then, the cells were collected and seeded into a 6-well plate at 1000 cells per well. After 10 days in culture, colonies were counted after fixing with 4% paraformaldehyde and staining with a crystal violet staining solution (Beyotime).

### Western blot analysis

T24 and UM-UC-3 cells were lysed with radio immunoprecipitation assay buffer (RIPA, Beyotime) supplemented with 1 mM phenylmethyl sulfonylfluoride (PMSF, Beyotime) after treatment, and then total proteins were extracted at 4 °C. Proteins were separated by sodium dodecyl sulfate polyacrylamide gel electrophoresis (SDS-PAGE) and then transferred to polyvinylidene difluoride membranes (PVDF, Millipore, USA). The membranes were soaked in 5% milk in TBS-T (Tris-buffered saline and 1% Tween 20) and incubated with primary antibodies in diluent overnight at 4 °C. The membranes were then probed with goat anti-rabbit IgG-HRP secondary antibody (EarthOx, USA) for 1 h.

### Statistical analysis

CCK-8, Caspase-Glo 3/7, mitochondrial membrane potential and ATP assays were performed four times each; ROS and apoptosis assays were performed three times each; GSH/GSSG and superoxide analyses were performed five times each; and cytotoxicity assays were performed six times. The results are presented as the mean ± standard deviation (SD). All data were analyzed using one-way ANOVA (SPSS (Statistical Package for the Social Sciences) 18.0). Differences were assessed using a Fisher’s least significant difference test [LSD (L)], and a significant difference was inferred when *P* < 0.05. An extremely significant difference was inferred when *P* < 0.01.

## Additional Information

**How to cite this article**: Qiu, M. *et al*. A reactive oxygen species activation mechanism contributes to JS-K-induced apoptosis in human bladder cancer cells. *Sci. Rep*. **5**, 15104; doi: 10.1038/srep15104 (2015).

## Supplementary Material

Supplementary Information
