# Supplementary material for: A reactive oxygen species activation mechanism contributes to JS-K-induced apoptosis in human bladder cancer cells
Source: Sci Rep. 2015 Oct 13;5:15104. doi: 10.1038/srep15104 (PMC4602210; doi:10.1038/srep15104)
Supplement: Supplementary Information [file srep15104-s1.pdf]

A reactive oxygen species activation mechanism contributes to JS-K-induced apoptosis in human bladder cancer cells

Mingning Qiu[1], Lieqian Chen[1], Guobin Tan, Longzhi Ke, Sai Zhang, Hege Chen*, Jianjun Liu**

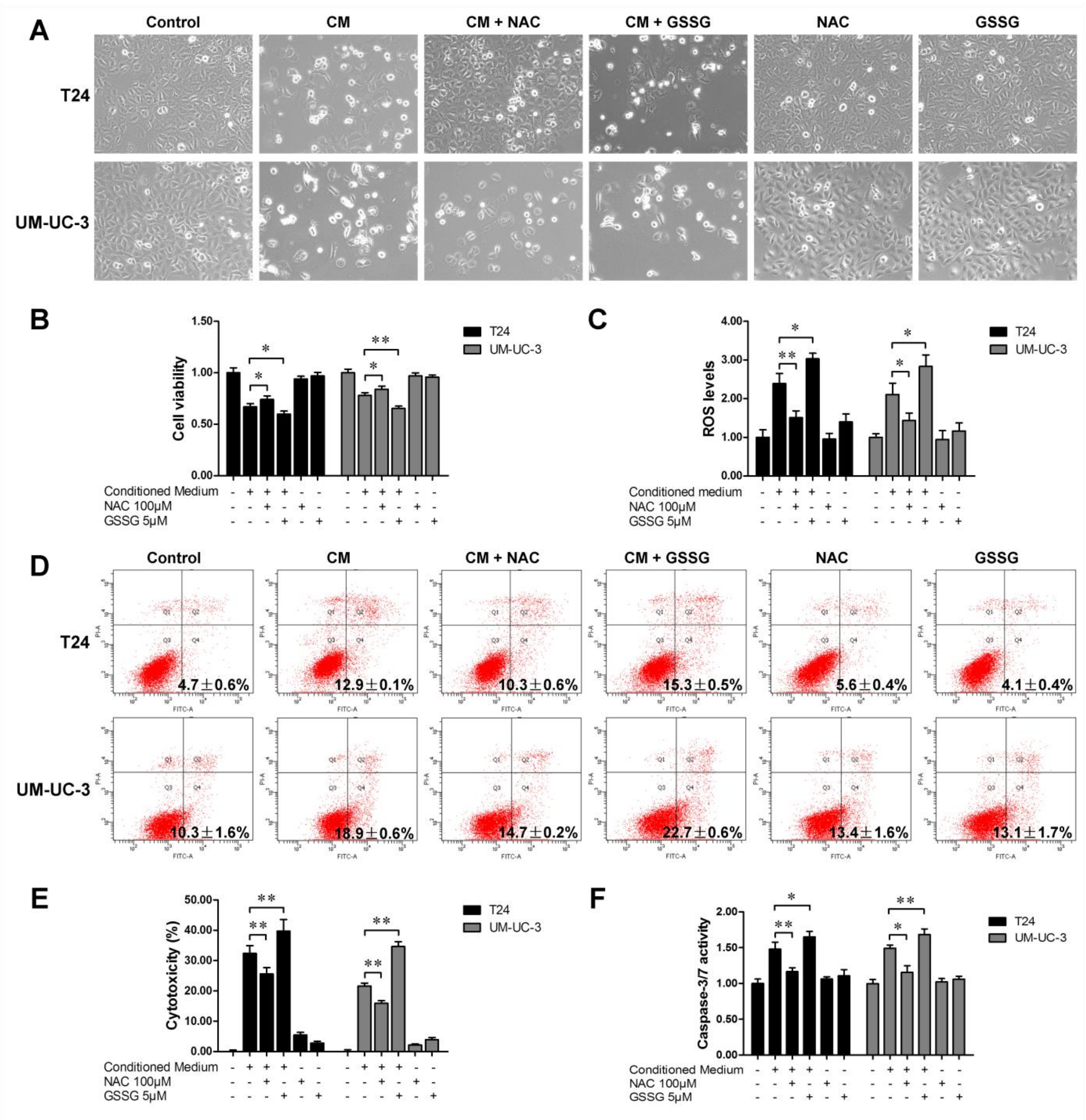

Figure S1. Effects of NAC and GSSG on conditioned medium-induced cell growth suppression and apoptosis

(A) Influence of conditioned medium on apoptosis in T24 and UM-UC-3 cells. Cells were incubated with 100 μM NAC or 5 μM GSSG for 24 h and then incubated with or without conditioned medium from cells treated with JS-K (5 μM). The cells were visualized by microscopy (100×). (B) Cell proliferation assay. (C) Cells were cultured in conditioned medium for 6 h after being pretreated with 100 μM NAC or 5 μM GSSG for 24 h, and the ROS level was analyzed. Apoptosis (D), cytotoxicity (E) and caspase-3/7 activity (F) were examined according to the distributions for the treatments in (A). The data are presented as the mean ± SD for at least three independent experiments. Single asterisks (*) indicate a significant difference ($P < 0.05$), and double asterisks (**) indicate an extremely significant difference ($P < 0.01$).

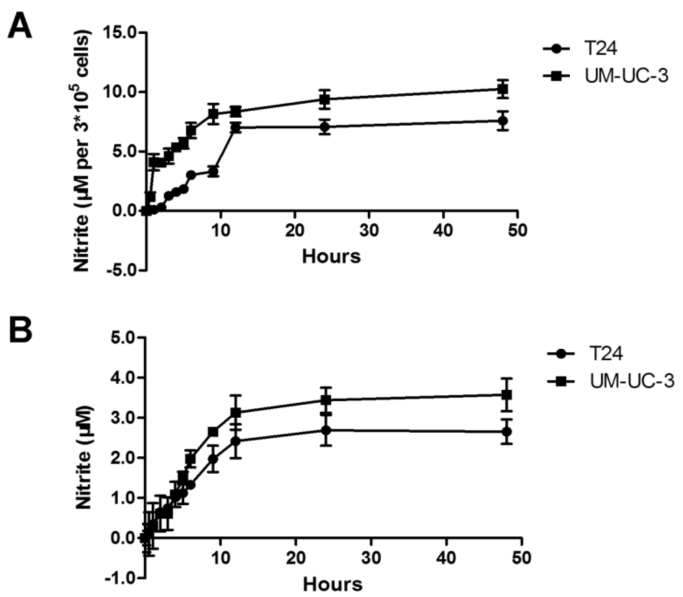

Figure S2. Release of NO from JS-K

Production of NO from JS-K in bladder cancer cells (A) and the medium containing 10 % FBS (B) was detected indirectly by detection of nitrite by using a NO assay kit (Beyotime). The data presented are the mean ± SD for at least three independent experiments.

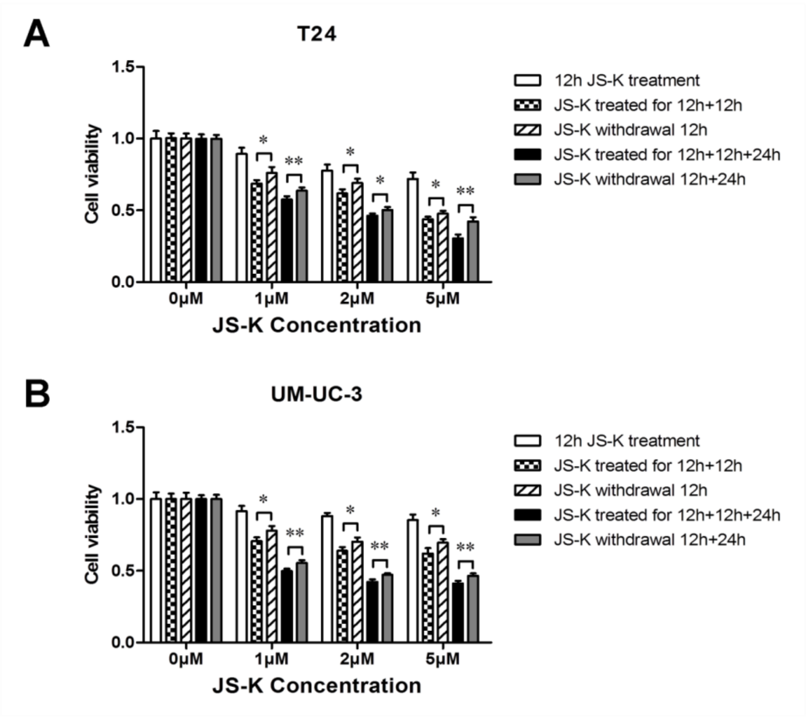

Figure S3. Bladder cancer cell proliferation with JS-K or JS-K withdrawal

T24 (A) and UM-UC-3 (B) cells were cultured with fresh medium up to 36 h after JS-K withdrawal after being treated with JS-K for 12 h. Cell viability was measured by a CCK-8 assay. The data presented are the mean ± SD for at least three independent experiments. Single asterisks (*) indicate a significant difference ($P < 0.05$), and double asterisks (**) indicates an extremely significant difference ($P < 0.01$).
